# ‘There's Nothing Wrong With You; You Just Need to Lose Weight’—A Qualitative Exploration of Pelvic Floor Dysfunction Among Women With Multiple Sclerosis and Their Interaction in Seeking Pelvic Healthcare

**DOI:** 10.1111/hex.14152

**Published:** 2024-07-15

**Authors:** Christine Addington, Andy Bradshaw, Suzanne Hagen, Doreen McClurg

**Affiliations:** ^1^ Department of Physiotherapy and Paramedicine Glasgow Caledonian University Glasgow UK; ^2^ Cicely Saunders Institute of Palliative Care, Policy and Rehabilitation King's College London London UK; ^3^ Nursing, Midwifery and Allied Health Professions Research Unit Glasgow Caledonian University Glasgow UK

**Keywords:** intersectional feminism, multiple sclerosis, participatory research, pelvic floor dysfunction, qualitative methods

## Abstract

**Introduction:**

Within 10 years of multiple sclerosis (MS) progression, nearly all women will have experienced symptoms associated with bladder, bowel and/or sexual health. Yet despite the impact these symptoms have on physical, psychological and social well‐being, it remains an underserved area within the UK healthcare system.

**Study Aim:**

This research employs a participatory research approach framed within the principles of intersectional feminism to collaboratively investigate the lived experiences of pelvic floor dysfunction (PFD) and healthcare interactions among UK‐based women with MS.

**Setting and Participants:**

Women residing in the United Kingdom with MS were invited to participate in online interviews facilitated by the primary author.

**Analysis:**

A thematic framework analysis offering a structured yet adaptable approach to data collection and interpretation.

**Results:**

One focus group involving four women with MS and seven individual, one‐to‐one interviews with women with MS provided insights into the challenges associated with navigating both MS and PFD. Four main themes included: Navigating MS and PFD; Cycles of Control; Mind, Mobility and Bladder Embodiment; Silenced Voices: The Impact of Taboos/Stigma/Dismissal on Preventing Access and Resistance through Collective Community. Six subthemes were also identified. Taken together, these themes cumulatively reflect PFD as an unmet healthcare need.

**Conclusion:**

Our findings underscore negative healthcare experiences, inadequate information provision and unmet needs related to PFD, emphasising the compounding effects of gender and disability biases.

**Impact:**

We hope that these insights can lay the groundwork for developing tailored therapeutic interventions and improved PFD healthcare for women with MS. Potential solutions include using existing MS support communities.

**Public Contributions:**

Women with MS were actively involved in co‐producing interview scripts for one‐to‐one interviews. The primary author shared study findings at an MS group event, engaging in discussions with over 30 individuals, including people with MS and their loved ones. MS advocates played a pivotal role in contextualising the study within the broader lived experience of MS.

## 1 Background

Multiple sclerosis (MS) is a disease affecting the central nervous system (CNS); current estimates suggest that 2.8 million people are globally living with MS [1], with women accounting for two‐thirds of all diagnoses [2]. MS is characterised by an immune‐mediated loss of myelin, the protective sheath that covers neurones within the CNS, helping transmit signals between brain and body systems. Over time, multiple scars (sclerosis) and neurodegeneration lead to both transient and progressive body system dysfunction [3]. As a result, individuals with MS may encounter a wide range of system dysfunctions owing to alterations in muscle control, coordination, cognition and fatigue [4]. Loss of muscle strength/endurance, coordination or control can lead to dysfunction among the muscles of the pelvic floor (PFD)—which can be associated with symptoms of bladder, bowel and sexual health (and wellbeing). Feelings of vulnerability and shame relating to these symptoms can lead to social isolation [5], withdrawal from relationships [6] and affect self‐identity [7]. PFD thus has a major negative impact on quality of life, with associated systemic implications given the costs of containment products [8]. Despite its profound impact, evidence‐based treatments for PFD in women with MS are limited [9], and access to these treatments remains unclear.

PFD can contribute to urinary and faecal incontinence, urinary retention, constipation and anorgasmia [10]. The incidence of PFD increases over time, especially in women and people assigned female at birth due to life events, such as pregnancy, childbirth, age and menopause [11]. Women with MS face an increased risk of PFD due to disruptions in neuronal communication between the CNS and the pelvic floor muscles, as well as associated organs, such as the genitals, bowel and bladder [12]. Due to the close anatomical proximity and shared neural pathways of these organs, it is not uncommon for symptoms to co‐occur, with almost all women with MS reporting some form of PFD within 10 years of diagnosis [13]. More specifically, data suggests that up to 80% of women with MS experience bladder and sexual dysfunction [14, 15] and around 50% face bowel function symptoms [16]. Despite these high incidences, research has shown that in comparison to men with MS, women are dissatisfied with the therapeutic management of symptoms of PFD, in particular sexual health [17]. This points to a gender‐based disparity in healthcare experiences and potentially reduced access to services that do not meet the needs of women with MS.

Pelvic floor muscle training (PFMT) is used in physiotherapy to treat, manage and prevent symptoms of PFD [18]. Despite global and UK government recognition of the importance of women being able to access pelvic health physiotherapy, challenges in access persist [19]; and can be exacerbated for women with disabilities such as MS [20]. Recent NICE [21] recommendations of PFMT as a first‐line treatment in conservative management of PFD have been based on studies that have excluded women with MS from participation‐causing concerns on the applicability of such interventions. Specific, NICE [9] neurogenic guidelines are limited to treating stress urinary incontinence, and had failed to be updated from 2012 to 2019 due to a lack of research. Thus current guidelines fail to capture the complexity of PFD treatment for women with MS. It is therefore plausible that a lack of evidence‐based treatment options would lead to limited patient−healthcare worker interactions. Woodward et al. [22] suggest that healthcare professionals may not proactively enquire about these symptoms. Indeed, as few as 2.2% of women with MS have had symptoms of PFD discussed with them by a healthcare practitioner [23]. Consequently, women with MS may struggle to access specialist physiotherapy for pelvic healthcare.

Access to healthcare may also be influenced by sociocultural norms and values, which, through reciprocal interaction, can shape healthcare research agendas [24]. These dynamics influence healthcare interventions and health professional training. Historically, topics on female sexuality, continence and genital health have been stigmatised, seen as taboo, shameful, or, in the case of sexual pleasure, unimportant for women [25], leading to neglect in medical priority and funding. In the United Kingdom, it has been estimated that less than 7% of medical research funds have been allocated to women's health since 2014 [26]. This underfunding can contribute to ongoing dismissal and gaps in women's healthcare, such as the misattribution of urinary incontinence as an inevitability of female ageing [27].

For women with MS, gender biases can be compounded by disability bias, leading to exacerbation in healthcare inequalities. In the field of MS, less than 10% of women's health studies focus on sex and intimacy interventions [28]. Further, in a recent Cochrane review on information provision in MS, none of the interventions contained information on sexual health [29]. Clinical education and healthcare interventions must be based on evidence‐based practice. Without adequate funding for a robust research base, healthcare professionals may lack appropriate resources or knowledge of existing healthcare pathways, resulting in a decreased ability to exchange information adequately during clinical appointments [30]. This may partly account for the reported lack of confidence healthcare professionals feel in treating symptoms associated with PFD, such as bladder dysfunction [31]. The lack of research and prioritisation diminishes treatment options, reduces clinical confidence in treating and thus impacts comprehensive care.

Comprehensive care relies on the development of interventions that have been evaluated by robust research methods, which can, in turn, help physiotherapists select effective interventions. However, patient satisfaction and adherence to interventions can diminish when interventions overlook the experiences and priorities of those most affected [32], such as women with MS and PFD. Incorporating women's perspectives through a patient‐centred approach can lead to better‐targeted treatments by understanding and incorporating specific challenges and needs into intervention development. Although our understanding of chronic illness has evolved with seminal works like Bury's theory of biographical disruption (how illness can impact a person's daily living and affect their identity) [33], it is critical to acknowledge that health experiences are influenced by multiple dimensions/demographic characteristics [34], including gender. The experience of PFD and accessing its care is influenced by social and cultural factors, making it a gendered experience that remains underexplored.

Despite increasing focus on PFD among individuals with MS [35], research into the lived experiences of women in the United Kingdom is sparse. This study uses a feminist lens and participatory research methods to explore how women with MS experience PFD and its healthcare management. By combining these approaches, we hope to promote patient‐centred care in the NHS and reduce the likelihood of inappropriate interventions and recommendations [36].

### 1.1 Aim

This study explored both how women with MS navigate their experiences with PFD and understand their interactions with healthcare services in addressing related symptoms.

## 2 Methods

Our study was guided by intersectional feminist and participatory research approaches. In the context of this study, intersectional feminism recognised that women's experiences of MS and PFD were the product of the complex interplay between personal (e.g., age, class, race, disability, sexuality, etc.) and structural factors (e.g., the often oppressive broader political, economic, social, and institutional contexts in which women live) [37]. This approach works harmoniously with participatory approaches by seeking to conduct research *with* and *by*—as opposed to *on* and *to*—participants, aiming to shift historic power imbalances often present in healthcare research [38]. Combining intersectional feminism with participatory approaches involves working in equal partnership to prioritise the voices and experiences of underserved groups historically excluded from research, practice and policy [39].

### 2.1 Study Design

We undertook a qualitative study using a combination of online video focus groups and semi‐structured online video individual interviews. The one‐to‐one interview script was developed from the online focus groups and later acted as the initial framework for thematic analysis.

### 2.2 Recruitment and Setting

Due to restrictions placed during Covid‐19, this study was based online. After receiving feedback from the focus group on the usability of Signal's open‐source messaging application for video conferencing, subsequent one‐to‐one interviews were conducted using the Microsoft Teams video conferencing platform.

To focus on the experiences of women within the context of the UK's healthcare system, participation was limited to people living within the United Kingdom. Participants were limited to cis‐gender women or people with anatomy assigned female at birth with a clinical diagnosis of MS and experiencing symptoms of PFD. PFD could include issues relating to bladder, bowel and sexual health but did not have to be clinically established.

The project used both snowballing and purposive sampling methods. Recruitment began with the support of a local MS Specialist Nurse. This individual contacted women with MS on their caseload who were known to be experiencing symptoms of PFD. To widen our reach, we also promoted the study via MS charity and MS community social media sites (see File S4). Informed consent was gained before arranging focus groups and one‐to‐one interviews. (Please see Files S1 and S3).

### 2.3 Data Collection

To facilitate meaningful one‐to‐one interviews, an interview guide was co‐created with four women with MS, during an online focus group. This participatory approach allowed for the developed interview questions to be centred around topics and issues that these women considered important. The four women took part in one 45‐min session, and two of these women then took part in another 45‐min session for final feedback on the proposed questions.

A further seven women diagnosed with MS participated in a single, individual online interview, lasting 45–60 min. Before their one‐to‐one interview, participants received examples of questions from the interview guide to allow time for self‐reflection and preparation of thoughtful answers to bring to interviews. The interview guide was centred around four themes: awareness of and access to pelvic healthcare, preference for pelvic healthcare, experience of MS and experience of PFD (see interview guide in File S2). An optional demographic questionnaire was also provided.

Online video interviews were arranged. Before the session, participants agreed to share their camera, created a pseudonym and were provided with a session agenda. Interviews were audio and video recorded, anonymised and transcribed verbatim.

### 2.4 Analysis

Audio data were analysed in NVivo 12 using framework analysis, chosen for its applicability in healthcare research and ability to explore relevant data, such as researcher notes, team discussions and reflections [40]. The method allowed for exploration within and between participants' experiences [41]. Analysis followed the seven steps of the framework analysis method [40]:

1. Familiarisation: Through re‐reading transcripts and re‐listening to video interviews. During this process, notes were taken on areas of interest, including fundamental issues raised, emotional responses, voice tone and the primary researcher's ‘analytic hunches’ (i.e., initial impressions of the data) [42]. Each written transcript was verified against the video for accuracy.
2. Coding: Labelling relevant segments of transcripts that aligned with our research aims.
3. Development of an initial analytical framework: Grouping similar codes into categories and categories into themes.
4. Indexing: Applying the initial framework back to raw data and refining it where appropriate.
5. Charting data: Data were categorised into charts and arranged to create order, help synthesise and create codes that reflected the data under each category. Hand‐drawn mind maps were used to explore the relationships between these categories.
6. Description: Defining and describing themes (and sub‐themes).
7. Interpretation of data as part of writing up the study findings.

### 2.5 Ethical Considerations

Institutional ethical approval (HLS/PSWAHS/20/028) was granted by Glasgow Caledonian University Health and Life Sciences Ethics, Nursing and Community Health Committee. Qualitative inquiry can often present subtle and unexpected ethical dilemmas unforeseeable by Research Ethics Committees. As such, we adopted an ‘ethics in practice’ approach [43] in which we recognised and treated ethics as an ongoing process. Specifically, to align our ethical approach and research design, we adopted a ‘Culturally Responsive Relational Reflexive’ approach to ethics whilst in the field [44]. This approach is: (i) *culturally responsive* by being sensitive to and accommodating for the cultural background and differences of participants, (ii) *relational* through ensuring that relationships between researchers and participants are grounded in mutual respect, dignity and connectedness, and, (iii) *reflexive* through reflecting individually, interpersonally and contextually to avoid power imbalances and support the autonomy, well‐being and safety of participants [44].

### 2.6 Rigour

The project followed the Standards for Reporting Qualitative Research (SRQR). However, it was acknowledged that an uncritical checklist does not guarantee a study's rigour [45], and so adopted the criteria from the TAPUPAS framework [46]. Integral to the study where steps such as member‐checking (participant feedback), developing a co‐produced interview script (e.g., increased applicability), positionality and reflexivity, seeking perspectives from supervisors, MS advocates and MS communities (i.e., critical friends) to maximise the study's credibility [45].

#### 2.6.1 Critical Friends

Study participants, members of our wider PPI network, and the wider research team acted as ‘critical friends’ [47]. This entailed working collaboratively to develop study materials and provide thoughts and feedback during data interpretation and implementation of findings into future studies. All those who had been a part of the focus group and interviews were invited to a one‐to‐one session with the primary author 6 months after their interview. The goal was to confirm the applicability of the interpreted themes [48]. In addition to this, the primary author was invited to present the initial findings to an in‐person MS diversity event, attended by 30 people, including people with MS, friends and family. Those attending provided anonymous comments on specific issues relating to barriers and priorities in relation to pelvic health, which helped to further refine the thematic analysis.

#### 2.6.2 Reflexivity

Throughout the research journey, the primary researcher aimed to understand the dynamics between themselves and women with MS by engaging with positionality and reflexivity. This played a crucial role in grasping the wider social contexts in which participants' experiences of PFD were located. I (C.A.) aimed to recognise and examine personal and social factors influencing outcomes during interactions, data collection and analysis. Documenting these reflections in diary entries using Rolfe et al. [49] the reflective model enabled meaningful changes to take place (after reflecting on the lack of diversity within our co‐development workshop members, the primary author reached out to specific MS charities [younger people with MS and BIPOC groups] and individual ‘MS diversity advocates’ within the United Kingdom to help the project reach women with MS from communities typically underserved by research. A digital illustration was created with a local artist to aid in the promotion of the study. With permission, adverts were then uploaded on social media platforms of United Kingdom‐based MS charities, and necessary alterations in wording were changed to be inclusive of the community that was being reached [50]).

The primary researcher's reflections explored key questions about being the ‘other,’ considering aspects like being able‐bodied, White, cis‐gender, but also of being an ‘insider’ in the shared experiences of being female, navigating healthcare with an invisible condition and providing support for mental and physical health issues [51]. These refelctions were used to deepen insights into the participants' experiences of PFD [52].

## 3 Results

Four women with MS participated in an online focus group, and seven different women with MS participated in an individual online interview. All women were aged 25–65, identified as female, and had the same gender as their sex assigned at birth. Most participants had relapse remitting MS, but one had secondary progressive MS. Time with diagnosis ranged from 1.5 to 20 years.

The analysis offers insights into the challenges of living with both MS and PFD, depicted through four main themes and six subthemes (seen in Table 1). Taken together, these themes are a cumulative reflection of PFD as an unmet healthcare need, requiring improved access and awareness of pelvic health physiotherapy.

**Table 1:** Themes and subthemes.

	Themes	Subthemes
PFD, an unmet healthcare need	Navigating MS and PFD: Cycles of Control	PFD's Disruptive Echo
	Mind, Mobility and Bladder Embodiment	Physiotherapy's Preoccupation with Mobility
	Silenced Voices: The Impact of Taboo/Stigma/Dismissal on Preventing Access	Off‐Limits
		Relentless Self‐Advocacy
	Resistance Through Collective Community	

### 3.1 Navigating MS: Cycles of Control

Living with MS was often described by the women as a journey marked by disruption, leading to feelings of uncertainty relating to potential symptom presentation. This unpredictability affected their mental and physical well‐being:

[I] wasn't great for maybe. 4 or 5 years, I was very … I couldn't go to a shop. I couldn't go out. I felt anxious about everything … I was just obviously unaware of what was around the corner and stressed out with it.

Interview, Jamie (56–65)

To manage this unpredictability, many of the participants adopted protective mechanisms, both emotional and physical, stating that they had to ‘get on with it’ and ‘just [had] to keep moving’. Yet, as Jax reveals, these strategies could fall short:

You wake up every morning and you are like ‘bugger, I need to do another day here’. Because you're just … MS has got you and you always go ‘I've got this, MS dinnae have me, I've got this’. But no, you're in its clutches. You are totally vulnerable to it, it's so changeable. So, it's just so overwhelming because you're just waiting. ‘What is coming now, what next, what else am I getting hit with?’

Interview, Jax (36–45)

Jax's relationship with MS captures the transient sense of empowerment that comes from effective management strategies or treatments and the subsequent feelings of loss when symptoms worsen or coping mechanisms fail. Seeking information and connecting with others living with MS helped to foster a collective experience crucial to managing uncertainty. However, for Jamie, who has lived with MS for over three decades, this process evolved into a deeper need to separate her identity from the disease:

I used to be, believe it or not, a counsellor for newly diagnosed patients with the MS Society, but over the years, I started questioning whether I wanted my identity to be so intertwined with MS that I lost sight of who I was beyond the disease.

Interview, Jamie (56–65)

#### 3.1.1 Subtheme 1: PFD's Disruptive Echo

PFD and its symptoms added complexity to living with MS. Symptoms like urinary incontinence, bowel issues and sexual dysfunction intensified the challenges and unpredictability that the women with MS experienced:

It's just so depressing [speaking about symptoms which have affected sexual and bladder function] and very, very …. not the person that I normally am. It's like there's two sides to myself.

Interview, Jamie (56–65)

The journey towards maintaining a distinct identity separate from MS is fraught with challenges. Jamie's efforts to maintain her identity separate from her disease were halted by PFD. For the women with MS, bladder, bowel and sexual function symptoms acted as an unwelcome reminder of having MS.

In comparison to MS symptoms related to mobility, the management strategies adopted by the women for PFD differed from the transformative impact of mobility aids, often described as ‘life‐changing’ [Jamie, 55–65]. Lack of access to specialised PFD healthcare and uncertainty of its availability led to restrictive self‐management strategies. These included either avoidance or meticulous planning, such as not attended social situations, creation of toilet location maps and reliance on incontinence pads—‘eliminating spontaneity from sex and intimacy’. [Focus group, Leigh, 55–65]. The fear of not finding a toilet was exacerbated by the lockdown of the COVID‐19 pandemic lockdown, as the closure of public toilets prevented some from leaving the house for exercise ‘I haven't been out of the house for a year’ Teagan (46–55). Systemic barriers, as explored in subsequent themes, highlight a critical gap in healthcare in supporting PFD symptoms for women with MS.

#### 3.1.2 Mind, Mobility, and Bladder Embodiment

Participants spoke of how PFD symptoms affected their whole body, discussing the multi‐directional relationship between their symptoms, mobility and mind:

Because if I need a pee my whole body goes ‘No, you're not going anywhere’ because obviously my brain's telling my bladder to hold onto the urine that's in there. But unfortunately, my brain's also telling the rest of my body [to hold on too], so as well as the manipulation of external body parts … in your mind you probably also have to go ‘right, let go, relax a wee bit’.

Interview, Jax (36–45)

Despite a lack of explicit knowledge of how physiotherapy could aid PFD, many women reflected on the disconnect between the bodily feelings caused by these symptoms and the health information received.

When I think back to the very beginning … the bladder thing was one of the first indications. It was one of the first things to come up. So, it's kinda weird that it's [the pelvic floor] not mentioned more.

Focus group, Leigh (56–65)

### 3.2 Subtheme 2: Physiotherapy's Preoccupation With Mobility

The experiences shared by women with MS suggest a disconnect between their embodied awareness of PFD and pelvic health physiotherapy availability. None had seen a specialist pelvic health physiotherapist or been offered physiotherapy for their PFD symptoms. There was some awareness of pelvic floor muscle exercises, although this was limited to those who had undergone pregnancy and childbirth. Despite bladder and bowel questions being a part of neurological and musculoskeletal physiotherapists’ subjective clinical assessments, the women saw physiotherapy as being preoccupied with mobility.

Taegan's experience illustrates this:

‘I've never seen a physiotherapist about the pelvic floor. They're really interested in your balance, or your walking. No physio has ever mentioned your bladder’. This statement highlights a lack of physiotherapy to recognise symptoms of PFD as an important part of their health.

Focus group, Teagan (46–55)

Leigh's uncertainty further emphasises this gap:

if there was a physio, I don't even know how that would work, and how it would be done.

Focus group, Leigh (56–65)

These insights characterise a resounding lack of awareness of the benefits of physiotherapy for PFD. Furthermore, knowledge of pelvic floor exercises was mostly limited to women who had been informed during pregnancy and childbirth, indicating a broader unmet educational need within the MS patient community.

When asked to envision what pelvic healthcare would look like, it was clear the women wished to have access to a team of healthcare professionals with an understanding of MS.

If there was an MS team [dedicated to pelvic health] they'd know to target these areas [bladder, bowel and sexual health], if not all of them. Physiotherapists or instructors [should] understand ‘right, this lady has MS, she's gonnae be working a wee bit differently so let's give her the patience to do so’.

Interview, Jax (36–45)

### 3.3 Silenced Voices: The Impact of Taboo/Stigma/Dismissal on Preventing Access

Prior negative healthcare experiences were among the many factors resulting in women with MS not wanting to raise their pelvic health concerns. The collective experience was that of dismissal, minimisation and failure to have their symptoms taken seriously by healthcare professionals.

The first time I went to see a bladder nurse they just told me, ‘there's nothing wrong with you, you need to lose weight’. That embarrassed me completely, so I never went back for years. That really put me off.

Focus group, Leigh (55–65)

#### 3.3.1 Subtheme 3: Off‐Limits

When trying to access pelvic healthcare, other participants experienced stigmatisation and unconscious biases from healthcare professionals surrounding women's health:

[quoting the bladder nurse] ‘I'm really thinking of changing my job because I can't spend all my time looking at women's mh‐mh [vulva/vagina].’ … I was really embarrassed to follow it up and after that I thought ‘Oh god it is embarrassing I don't really want you looking at my mh‐mh [vulva/vagina]’.

Taylor, Focus group (56–65)

This quote underscores the detrimental effects of problematic attitudes and biases within women's healthcare settings. Here, the nurse's words resulted in Taylor feeling that her symptoms were a burdensome, undesirable aspect of healthcare professionals' jobs. This caused her to feel ashamed of these symptoms, which may have been intensified by the failure of the healthcare profession to use anatomically correct terminology. This incorrect terminology implied that the discussion of her condition was taboo, and the experience caused enough distress to prevent Taylor from seeking future care. The situation contrasts starkly with the resounding need to have healthcare professionals ask about PFD symptoms.

it's not something that I'd actually go and ask for help with … [like] ‘oh, when I have sex it's sore’, so I think yeah it would have to be something that would have to be directed to you [by the MS nurse].

Interview, Marin (36–45)

Marin's quote shows the importance of trusted healthcare professionals initiating conversations with women experiencing symptoms of PFD, as these women may not feel comfortable raising these issues themselves or know that help is available.

#### 3.3.2 Subtheme 4: Relentless Self‐Advocacy

The women with MS described their persistence in bringing attention to specific aspects of their health condition in the face of continual dismissal.

Robin (25–35)

Initially, the theme was described as self‐advocacy. However, during the member check process, Robin (25–35) suggested that it should be prefaced with ‘relentless’. She wanted to make it clear to readers that such advocacy takes a profound physical and emotional toll via the constant need to validate and explain her symptoms.

So I think most of my friends with MS' attitude is that you have to be a bit pushy. Someone was saying: ‘I finally got to speak to a fatigue specialist’, and everyone was like ‘I didn't even know that was a thing’.

Interview, Robyn (25–35)

### 3.4 Theme 4: Resistance Through Collective Community

Participants highlighted the challenges of navigating a complex healthcare system, often without a full understanding of its intricacies and terminology. MS‐specific groups were perceived as valuable as they provided a community of shared MS experiences in which bladder, bowel and sexual health symptoms were validated.

every woman with MS that I've spoken to has issues in some of these areas [bladder, bowel and sexual health], if not all of them.

Interview, Jax (36–45)

Being part of a group with shared experiences provided women with MS a chance to openly discuss topics deemed ‘off limits’ in healthcare and social settings. This helped remove stigma and provided a place of empathy.

With regards to pelvic health, it's not really something that anyone's really mentioned from a healthcare professional point of view, it's more like the girls in the group chat will be like ‘I'm more likely to get a flare‐up or a relapse if I'm on my period’.

Interview, Robyn (25–35)

Community, both physical and virtual peer support, provided an opportunity for sharing information and emotional support. Such groups provided a space where participants could unmask without fear of judgement.

I never had a clue about [an MS support charity] That was a godsend because suddenly I was in with a group of people who all had MS. (…) and it was just like ‘oh my god, you can sit and just totally be at ease!’ Because, they just know, like nothing has to be explained. Nothing! (…) I can talk to them probably better than my close friends because not having MS, you just don't get it.

Interview, Jax (36–45)

## 4 Discussion

This study provides novel insight into how women with MS experience PFD and interact with healthcare services. For symptoms associated with PFD, women face healthcare biases, social taboos and a lack of understanding from professionals, which hinders their access to effective treatments like physiotherapy, leading to unmet PFD healthcare needs. The unmet healthcare need for PFD symptom support can exacerbate feelings of vulnerability present in navigating an unpredictable disease.

Insights from our study have highlighted the significance of having protective mechanisms in place to mitigate the impact of MS symptoms and prevent the disruption to personal narratives that Bury [33] first theorised. This aligns with wider research highlighting the importance of protective mechanisms in providing resilience to carry out their daily activities and thus preserving their sense of self [53]. Mobility devices allowed our participants to re‐establish routines that reinforced their identity. However, unlike the autonomy afforded by mobility aids, managing symptoms related to bladder, bowel and sexual health proved more challenging and acted as a reminder of their illness. We argue that this may partly be due to the lack of funding dedicated to women's health [26] and the historical de‐prioritisation of women's health in scientific discourse [25]. Without robust research, healthcare professionals may lack interventions for effective PFD management. Our participants, therefore, resulted to engaging in restrictive self‐management strategies, intensifying their feelings of vulnerability, as reported elsewhere [54].

The unmet need for PFD care, especially the sexual health needs of women, could reflect a broader societal failure to recognise them as sexual beings [55], a prejudice that also exists for people with disabilities [56]. This is reflected in the limited research available for PFD symptoms in MS [29]. Factors given importance in society are known to influence healthcare policy [24] and, in turn, shape the education of healthcare practitioners and their clinical practice. Physiotherapy education has been historically based on the biomedical model, centred on ‘fixing’ the bodies of those with disabilities through rehabilitation or the maintenance of limb motor control and function [57]. Such emphasis during clinical education may result in physiotherapists feeling equipped to aid mobility but less confident in discussing symptoms associated with sexual health, bladder or bowel [58].

Unintentional biases present in healthcare training not only undermine the understanding of women's health needs but may also exacerbate societal stigma. Stigma is not only present at the level of the system but, as seen in our study, exists also at the level of the individual healthcare worker. In our study, the reluctance of a healthcare professional to conduct vaginal examinations, viewing it as an undesirable task, mirrors broader systemic issues in the healthcare field. This attitude reflects the research of Stewart [59] and Bolton [60], who highlighted the stigmatisation surrounding intimate health and the perception of women's bodies as contaminated or dirty. Such perspectives can lead women with MS to internalise this stigma, causing shame and embarrassment preventing access to appropriate healthcare for PFD.

Other systemic factors may have acted to prevent our participants from accessing PFD support. The self‐management model in healthcare, particularly for women with MS dealing with PFD, may unintentionally hinder access to necessary services. This model requires patients to initiate discussions about their PFD symptoms [22]. However, effective self‐advocacy in health depends on adequate health literacy, specifically understanding female genital anatomy and body functions. This level of health literacy contrasts with the realities shown in a recent UK survey, in which only 9% of women could accurately identify components of external female genitalia [61]. Even those with high levels of health literacy can find the healthcare system challenging to navigate when seeking PFD help [62]. Accordingly, our findings reflect reduced confidence in women effectively communicating with healthcare providers.

In addition to the reduced confidence in navigating the complexity of healthcare, their experience of dismissal/invalidation when trying to seek support acted to further prevent access to appropriate health pathways. In our study, one woman was told there was nothing wrong with her other than needing to lose weight. Like other studies' insights on women's experience of barriers in accessing healthcare [63], the women in this study's PFD symptoms had been inappropriately attributed to weight. Continued dismissal requires women to continuously advocate for their health needs [64]. Doing so with a disability—such as MS—has a compounding effect, as symptoms of brain fog and fatigue may mean reduced energy availability, resulting in an increased emotional and energetic burden. The consequence, described in the literature, is that individuals with MS tend to defer healthcare interactions until they are confronted with what is described as a health crisis [65].

Failure to seek or access support for PFD could result in serious consequences, as symptoms may progress beyond the point where conservative approaches such as physiotherapy are not effective [66]. Our study suggests that women need to be asked about PFD symptoms, but healthcare professionals have not instigated discussions. This is echoed in other groups seeking PFD support, such as antenatal care [67]. Gatekeepers, such as MS nurses, could help overcome this problem by providing vital information on services, hence facilitating women to navigate their healthcare needs. However, the workload for these healthcare professionals, who are often individually responsible for over 300 individuals [68] could reduce their ability to cater to service users' individual, multifaceted needs. A multidisciplinary approach may allow for other healthcare professionals, physiotherapists in pelvic health and pharmacists to work alongside the MS Nurse, to reduce the workload and ensure that all patient needs can be met.

### 4.1 Solutions: Community Pelvic Healthcare and Upskilling of Healthcare Professionals

Our findings demonstrate that the healthcare system's neglect of bladder, bowel and sexual health symptoms resulted in women seeking validation of their symptoms from external sources. This was mainly in the form of peer support, fostered through online MS communities or local MS charitable services. While these support groups have been incredibly valuable, our participants still struggled to comprehend how to manage symptoms of PFD or how pelvic health physiotherapy could help. Which is not surprising given the UK's poor health literacy in women's health [61]. A solution might be to work alongside already established groups to create health promotion community action interventions.

For example, Almeida et al. [69] created a community health promotion intervention using a textile toolkit to educate women on the form and function of their genitalia and pelvic floor muscles. Body mapping activities and wearable textiles helped facilitate more accurate sharing of information and sparked women's curiosity to learn more [69]. Storytelling and dance [70] and ‘do‐it‐yourself’ science demonstrations [71] have also been used as community engagement activities as mediums through which people can share experiences, feel connected and potentially manage their symptoms. Thus, community‐based engagement has been shown to have a positive impact on health‐related behaviour change [72]. Such community‐based interventions could help improve access to pelvic healthcare and improve awareness of self‐management/prevention of PFD.

### 4.2 Strengths and Limitations

A strength of this study was the participatory approach through which we ensured that people with lived experience were involved in all stages of the research process. This means our research is likely to be more impactful by focusing on topics that are important to women with MS‐related PFD symptoms, alongside representing them in ways that are sensitive and accurate to their experiences. As such, these findings are more likely to have naturalistic generalisability. That is, the findings of this study are likely to resonate with the personal experiences of other women who have PFD symptoms [47].

A major critique of our study was that only White Scottish/British women with MS were interviewed; the experiences of women of colour were absent, leading to a healthcare narrative that primarily reflects White experiences [73]. This gap can result in healthcare services that do not fully cater to the needs of women of colour with MS. Additionally, the research did not consider factors like the length of time since MS diagnosis, the type of MS or the participants' ages, all of which could influence healthcare needs. Understanding these nuances will be essential for tailoring healthcare services to the diverse circumstances of individuals with MS.

## 5 Conclusion

Living with MS is characterised by uncertainty and disruption. This project has explored the often‐overlooked challenges faced by women with MS and PFD. Our studies insights suggest that symptoms of PFD may act as a reminder of the vulnerability of living with MS. We argue that, unlike other aspects of symptom management, challenges persist in accessing suitable PFD healthcare due to limited awareness of physiotherapy benefits, negative healthcare experiences and systemic biases, leading to restrictive self‐management practices. In resistance to these unmet needs, already existing MS networks could provide an opportunity for community‐based interventions for women living with MS. This study offers insights that may improve healthcare equity for women with MS and PFD in the United Kingdom. However, continued research involving a diverse range of participants is needed to enhance the care and support provided to all people with MS.

## Author Contributions

**Christine Addington:** conceptualisation, investigation, writing–original draft, writing–review and editing, methodology, formal analysis, software, project administration. **Andy Bradshaw:** methodology, writing–review and editing, formal analysis, supervision. **Suzanne Hagen:** supervision, formal analysis, writing–review and editing, methodology. **Doreen McClurg:** methodology, writing–review and editing, supervision, formal analysis.

## Ethics Statement

Institutional ethical approval (HLS/PSWAHS/20/028) was granted by Glasgow Caledonian University. Participants were informed about voluntary participation and their right to withdraw from the interview at any time. The study's aims were provided, and all individuals who expressed interest in the study had the opportunity to ask any questions before consenting to participate.

## Consent

Informed E‐consent was obtained from the participants before the interview. The interview was conducted according to the time and preferences of the participants.

## Conflicts of Interest

The authors declare no conflicts of interest.

## Supporting information

Supporting information.

Supporting information.

Supporting information.

Supporting information.

## Data Availability

The data are not publicly available due to privacy and ethical restrictions. Upon reasonable request, the de‐identified data supporting this study's findings are available from the corresponding author.
