# Supplementary material for: ‘There's Nothing Wrong With You; You Just Need to Lose Weight’—A Qualitative Exploration of Pelvic Floor Dysfunction Among Women With Multiple Sclerosis and Their Interaction in Seeking Pelvic Healthcare
Source: Health Expect. 2024 Jul 15;27(4):e14152. doi: 10.1111/hex.14152 (PMC11249810; doi:10.1111/hex.14152)
Supplement: Supplementary file 1 — Supporting information. [file HEX-27-e14152-s003.pdf]

Supplementary Information

CONSENT FORMS AND DEMOGRAPHIC SURVERY

Ethics Approved:
HLS/PSWAHS/20/028

Glasgow Caledonian University

'What women want…... insight into the lived experience of pelvic floor dysfunction in women with multiple sclerosis to improve physiotherapeutic intervention"

PPI
CONSENT FORM

Please initial box

1. I confirm that I have read and understood the information sheet dated 30/10/2020 (Version 1) for the above study and have had the opportunity to ask questions and have had these answered satisfactorily. ☐

2. I understand that my participation is voluntary and that I am free to withdraw at any time without giving any reason without my medical care or legal rights being affected. ☐

3. I understand that this focus group will be virtual and will involve me registering and downloading Signal a private and secure messenger. When you create a Signal account you register a phone number. Your calls and messages are always encrypted, so they can never be shared or viewed by anyone but yourself and the intended recipients. I have read and understood Signal's terms and privacy policy and agree to using Signal. ☐

4. I agree to take part in the focus group outlined in the Participant Information Sheet-focus group ☐

5. I understand that my participation will be audio-recorded, and analysed, anonymised quotes may be used in publications about the research however, it will not be possible to identify me from this information. I give my permission for this. ☐

6. I understand that the results from this will inform future work that is intended to be published however, it will not be possible to identify any participant from this. ☐

7. I agree to being sent a copy of the summary of the day. ☐

8. I agree to being contacted after the workshop about possible involvement in future work but understand that this is optional ☐

________________________ ____________
Date Initials

____________________________ ____________ ___________________________
Name of person taking consent Date Signature

Insight into the lived experience of pelvic floor dysfunction in women with multiple sclerosis to improve physiotherapeutic intervention

INTERVIEW
CONSENT FORM

Please initial box

1. I confirm that I have read and understood the information sheet dated (version 1) for the above study and have had the opportunity to ask questions and have had these answered satisfactorily.

2. I understand that my participation is voluntary and that I am free to withdraw at any time without giving any reason without my medical care or legal rights being affected.

3. I agree to take part in a virtual or telephone one-to-one interview outlined in information sheet dated …(version …)

4. I understand that consenting to a virtual interview will involve me registering and downloading Signal a private and secure messenger. When you create a Signal account you register a phone number. Your calls and messages are always encrypted, so they can never be shared or viewed by anyone but yourself and the intended recipients. I have read and understood Signal's terms and privacy policy and agree to using Signal.

5. I understand that my participation will be audio-recorded, and analysed, anonymised quotes may be used in publications about the research however, it will not be possible to identify me from this information. I give my permission for this.

6. I understand that the results from this will inform work that is intended to be published however, it will not be possible to identify any participant from this.

7. I agree to being sent a copy of the summary of the results once the study has been written up.

8. I agree to being contacted after the workshop about possible involvement in future work but understand that this is optional.

____________________ __________ ____________________

Initials of participant Date Signature or initials

____________________ __________ ____________________

Name of person taking consent Date Signature

PH Demographic Questionnaire

Pelvic Health Interview Demographic Questionnaire

As part of the study process, we will be asking you a few demographic questions; please share as much or as little as you feel comfortable. Inclusion and diversity are a key priority of this research study. We want to ensure that this study reflects the experiences and needs of the diverse population of individuals with MS living in the UK. In doing this we hope to develop a patient centred, co-produced physiotherapy programme.

You do not have to provide answers to anything you do not wish to disclose

Question Title

1. What is your age in years?
   Please leave blank if you would prefer not to answer. 0

Question Title

2. In what regional county do you currently reside?
   Please leave blank if you would prefer not to answer. 0

Question Title

3. How do you currently describe your race/ethnicity?
   Please leave blank if you would prefer not to answer. 0

Question Title

4. How do you currently describe your gender identity?
   Please leave blank if you would prefer not to answer. 0

Question Title

5. Is your gender the same as the sex registered to you at birth?
   Please leave blank if you would prefer not to answer. 0

- ☐ Yes
- ☐ No

Question Title

6. Currently, what pronouns do you identify with?
   Please leave blank if you would prefer not to answer. 0

Question Title

7. What is your current MS diagnosis?
   Please leave blank if you would prefer not to answer. 0

Question Title

8. Do you currently or have you in the past experienced issues with either bladder, bowel and/or sexual health? If so what forms of management have you tried/been offered?
Please leave blank if you would prefer not to answer. 0

Question Title

9. Do you currently use any assistive devices for mobilising around the house and or in the community? please leave blank if you would prefer not to answer 0

0 EDIT OPTIONS MOVE COPY DELETE

Question Title

10. If you have any feedback regarding this questionnaire please feel free to write below

Thank you for taking the time to do this questionnaire. It is greatly appreciated. 0
