# Supplementary material for: ‘There's Nothing Wrong With You; You Just Need to Lose Weight’—A Qualitative Exploration of Pelvic Floor Dysfunction Among Women With Multiple Sclerosis and Their Interaction in Seeking Pelvic Healthcare
Source: Health Expect. 2024 Jul 15;27(4):e14152. doi: 10.1111/hex.14152 (PMC11249810; doi:10.1111/hex.14152)
Supplement: Supplementary file 2 — Supporting information. [file HEX-27-e14152-s002.pdf]

Supplementary information

Focus Group and Interview Agendas

Focus Group Agenda

(The session should last no longer than 1hr 15 mins but to allow for any technical hitches etc. I have timetabled 1 hr 30 mins)

Materials required for the session: Paper, pens, a cuppa of your choice (optional!) and of course yourself!

What you can expect on the day……

Safe space:

It is my intention that this session be a safe space in which everyone can share as they feel comfortable. I understand that group discussions about sensitive topics, concerning bladder, bowel and sexual health can be difficult. We may feel embarrassed to speak up, but please be assured that this group will be conducted free from judgment. So that you all feel comfortable in this session we will all agree to maintain the confidentiality of the information discussed by participants and researchers during the focus group session. Some ground rules to help us foster this safe space are below.

- We will help protect others' privacy by not discussing details outside the group.
- Know that it's ok to abstain from discussing specific topics if you are not comfortable. Speak only as openly as you feel comfortable.
- All responses are valid—there are no right or wrong answers.
- We will endeavour to devote our full attention to each person who speaks and allow each person to take their time and complete their thoughts.
- We will use people's pseudonyms and may refer back to earlier comments they've made.
- We can share our thoughts on what is valuable about someone's question or comment.
- Please respect the opinions of others even if you don't agree
- Please remember that participation in the focus group is voluntary, you may at any time withdraw from conversation without reason.

If there is anything you would like to add to this list please let me know via email or at the start of the session. I will provide space to share.

Welcome and background (10mins): A warm welcome and a little about me and the project.

I will provide a very brief introduction on pelvic floor dysfunction associated with multiple sclerosis, typical symptoms of PFD and physiotherapeutic management.

Warm-up (10 mins):

Before we start the discussion, we will have a small warm-up. If you all feel comfortable, we will do two warm-up exercises

1. What do we have in common: Find 10 things in the group that you all have in common?
2. One word that describes your experience of pelvic floor dysfunction and then another word to describe your experience of physiotherapy and pelvic floor symptoms (bladder, bowel or sexual health

Potential questions 30mins:

Before the session I invite you to think about your answers to the following questions…..

1. What is your experience of physiotherapy?
2. How do you imagine physiotherapy can help pelvic floor symptoms?
3. Imagine that you are being invited to an interview to be asked about your experience of PFD, what would you expect to be asked?
4. Imagine that you had the perfect physiotherapy programme, how would it improve your quality of life (physical and psychological)?

Closing the session and a big thank you (10 mins)!

To end the discussion, we will summarise the main points. You will be invited to reflect on the main ideas and asked if you have any additional thoughts to share.

Afterwards, I will analyse the pseudonymized discussion to develop an interview script, no names or identifiable data will be used of saved from this focus groups findings. If you would

like to be sent a copy of this script I would be happy to do so. Any feedback on the script will be gratefully received.

<u>Interview Agenda</u>

<u>Virtual Chat Agenda What you can expect on the day</u> (7-minute read)

The session should last no longer than 1hr but to allow for any technical hitches etc. I have timetabled 1 hr 30 mins.

Safe space:

To help inform pelvic health care, in this virtual chat I am going to be inviting you to share your experiences around bladder, bowel and sexual health. It is my intention that this session be a safe space in which you may share as you feel comfortable. I appreciate questions around bladder, bowel and sexual health can bring up strong physical feelings and emotions of discomfort and or trauma. Please know that you are in charge of this chat. As we go along, if there are any questions that you would rather not answer, this is fine, let me know and we will move on. At the end of the session, I will send you an email with a list of resources should you need further assistance.

To help foster a safe space during our virtual chat I have put together some guidelines:

- I will endeavour to devote my full attention to you, and will provide you with the time and space, you need to tell me your experiences.
- This will be a non-judgemental space. At times I may ask you to dive into your experience a little further. This is not to challenge your experience but so that I can try and gain a better understanding of your experience and needs from healthcare.
- To help protect your privacy, there will be no identifiable data attached to your interview.
- Please know that it’s ok to abstain from discussing specific topics. You do not have to provide reason. Speak only as openly as you feel comfortable.
- All responses are valid—there are no right or wrong answers.
- Please remember that your participation is voluntary. You may at any time withdraw from conversation without reason.

If there is anything you would like to add to this list, please let me know via email or at the start of the session. At the start of the virtual chat, I will provide space to share.

Welcome and background (10mins): A little about me and the project.

I will provide a very brief introduction on the anatomy of the pelvic floor, symptoms that can be associated with multiple sclerosis and the role of physiotherapy.

About you (10 mins): Before we start discussing the specifics around your pelvic health, I invite you to tell me a little about yourself.

- Could you describe your journey with MS? How does your MS present itself? What is your current relationship with your MS?
- What treatment/s have you received for MS?
- What is your living situation (i.e., where and with whom do you live with)?

In the next section we will be focusing on symptom relating to your pelvic floor muscles (Approx. 30 minutes of conversation):

Our pelvic floor or pelvic diaphragm consists of three muscle groups. These muscles are responsible for several vital functions. These include supporting our pelvic organs, stabilising our joints, assisting in urinary and faecal continence, sexual performance, facilitating the birthing process and they also help to circulate blood and lymph.

Putting myself in your shoes, what feelings and emotions would arise about my pelvic floor? And what symptoms would I be experiencing?

1. **How have issues with bladder, bowel and sexual health influenced you and your life?**

   A) Your physical well-being (i.e., pain, fatigue levels, mobility)?

   B) Psychological well-being (i.e., sense of control, self-esteem)?

   C) Social well-being (i.e., social interactions)?

   D) Sexual wellbeing (i.e. sexual pleasure, desire, sensation)

2. **What has your experience been of accessing pelvic floor healthcare?**

- How are you currently managing symptoms relating to bladder, bowel and or sexual health?
- Have you ever been asked by a physiotherapist about your pelvic health-if so, what was your experience?
- Have you ever had an internal examination for your pelvic floor muscles?

3. **How could physiotherapy assist you?**

- What role do you think a physiotherapist could have in assisting you with your pelvic healthcare? How would you like to be approached regarding pelvic health?
- Think back to your answer around quality of life, what aspects would you like physiotherapy to support you with.
- **How would you feel** about discussing your bowel, bladder, and sexual health, with a physiotherapist? What might make this conversation more comfortable?

4. **What challenges does physiotherapy present for you?**

- What aspects of your experience of MS are crucial when designed a pelvic floor programme?
- Are there any aspects of exercise that you find worrying?
- Do you think there would be any barriers (i.e., pain, fatigue, mood, mobility) that would prevent you from doing a pelvic healthcare programme?

5. **Are there any aspects of your experience that you think I have missed and would be integral to informing physiotherapy management?**

Closing the session and a big thank you (10 mins)!

To end the discussion, we will summarise the main points. You will be invited to reflect on the main ideas and asked if you have any additional thoughts to share. Afterwards, I will analyse your answers alongside other individuals with MS to find common themes and differences.

I am currently in the process of thinking up ways of ensuring that your voice is heard in pelvic healthcare and making sure that I am telling your story right. I would appreciate any suggestions on how best to do this. I want to make sure this research study is guided by you!
