# Supplementary material for: ‘There's Nothing Wrong With You; You Just Need to Lose Weight’—A Qualitative Exploration of Pelvic Floor Dysfunction Among Women With Multiple Sclerosis and Their Interaction in Seeking Pelvic Healthcare
Source: Health Expect. 2024 Jul 15;27(4):e14152. doi: 10.1111/hex.14152 (PMC11249810; doi:10.1111/hex.14152)
Supplement: Supplementary file 3 — Supporting information. [file HEX-27-e14152-s001.pdf]

Supplementary information:
Recruiting Advertisement

Let's Talk Pelvic Floor: Bladder, Bowel and Sexual Health in Women with Multiple Sclerosis

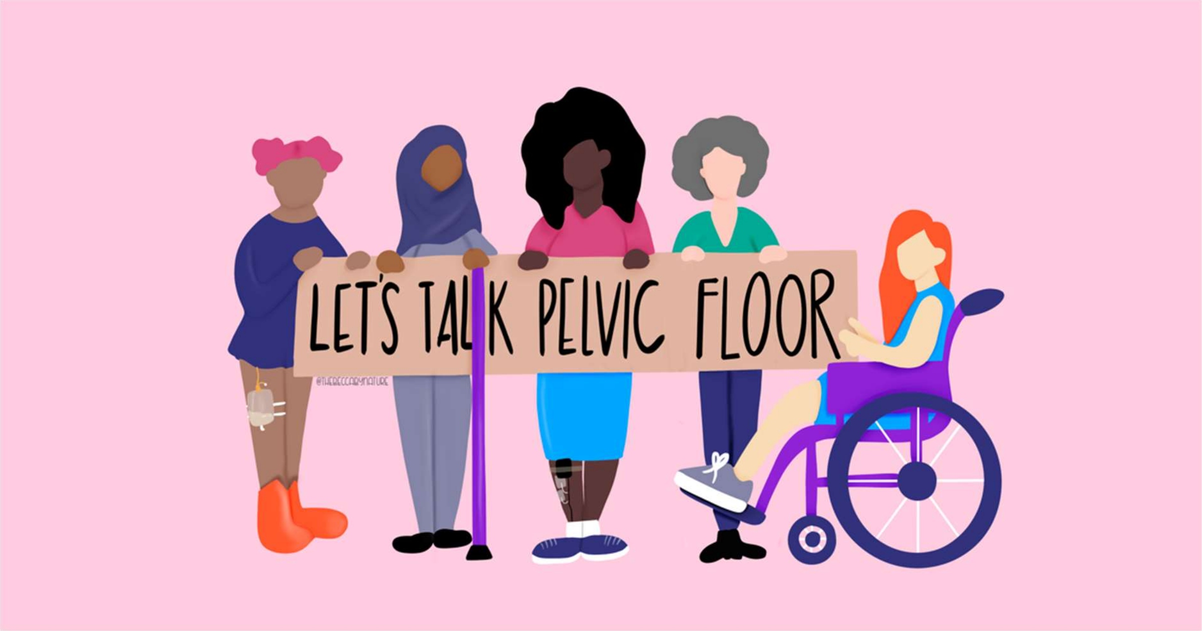

Over **50%** of women with MS will experience symptoms associated with pelvic floor function. These could include issues relating to bladder, bowel and sexual health. Unfortunately, bladder, bowel, and sexual health remain under-represented within both research and clinical practice.

To try to understand how physiotherapy can better serve your pelvic floor health, we are undertaking a research project in which we would like to hear about your experiences of living with MS and pelvic floor health concerns/symptoms.

If you would like to find out more about this research study, please contact Christine Addington; Email: caddin200@caledonian.ac.uk (Participants must be 18+)

Christine is a Pre-registration Physiotherapy student, who is exploring the lived experience of women with MS and symptoms related to pelvic floor function. This study will be the first in the UK to ask women with MS about their symptoms relating to pelvic floor health.

This physiotherapy research study is committed to diversity and inclusion.

Let's Talk Pelvic Floor digital illustration was created by illustrator @thebeccabynature for Christine Addington and her research project.
