# Supplementary material for: ‘There's Nothing Wrong With You; You Just Need to Lose Weight’—A Qualitative Exploration of Pelvic Floor Dysfunction Among Women With Multiple Sclerosis and Their Interaction in Seeking Pelvic Healthcare
Source: Health Expect. 2024 Jul 15;27(4):e14152. doi: 10.1111/hex.14152 (PMC11249810; doi:10.1111/hex.14152)
Supplement: Supplementary file 4 — Supporting information. [file HEX-27-e14152-s004.pdf]

Participant Information Forms

Let's talk pelvic floor …... insight into the lived experience of pelvic floor dysfunction in women with multiple sclerosis

Introduction and study purpose

Living with multiple sclerosis (MS) can cause symptoms connected to pelvic floor dysfunction. These symptoms may include urinary incontinence, issues with bladder emptying, pelvic organ prolapse, sexual dysfunction and chronic pelvic pain. Physiotherapy has the potential to improve pelvic floor dysfunction. It can improve muscle function, increase confidence and self-esteem, and enhance quality of life. However, for women with MS, current therapy guidelines for pelvic floor dysfunction are inadequate. This study will be one of the first in the UK to ask women with MS about their experiences and needs. The study will consist of three phases:

Phase one: a short online focus group;

Phase two: a private one-to-one interview; and

Phase three: the development of a physiotherapy programme.

You are being invited to take part in **Phase one**, which will consist of a short online focus group. Your answers will provide information in the area that is currently lacking, to help inform interviews in Phase two. At this focus group we will discuss your experiences of pelvic floor dysfunction and its management. During our focus group we will also concentrate on the potential of physiotherapy to improve symptoms of pelvic floor dysfunction. Your contribution will help us gain a greater understanding on the impact of pelvic floor dysfunction in women with MS. The aim of this focus group is to discuss areas that you think we need to include when conducting Phase two of the study. The outcome of the focus group will be the development an informed interview script. This interview script will be used in Phase two of the study. This study is being carried out by Christine Addington (student researcher) and is supervised by Professors Doreen McClurg and Suzanne Hagen at Glasgow Caledonian University and Dr Andy Bradshaw at Hull York Medical School. The study makes up a component of the student researcher's Doctorate in Pre-registration Physiotherapy award.

Before you decide whether to take part, it is important for you to understand what participation in this focus group will involve. Please take time to read the following information carefully and discuss it with others if you wish. Please contact us at the address below if you would like any more information.

What will I have to do if I take part?

If you are interested in taking part in this meeting, you will need to be able to access an electronic device with internet connection that supports video call software. You will be invited to attend an online group meeting using Signal, a secure video call programme. Details on how to use Signal will be provided by email. The meeting will last no longer than one hour and will be arranged for a date and time that is convenient for those involved. The meeting will include the research student Christine Addington, who will facilitate the meeting, and women with MS (4-6 individuals). You will be presented with brief information regarding the problem of pelvic floor dysfunction in MS and current physiotherapeutic treatments. You and the other members will then be asked to discuss your experiences of physiotherapeutic treatment options (if any), and the benefits and problems with these treatments, to try to identify a way forward for Phase two of the study. The overall aim is to create better evidence for patients and clinicians on how therapies can be improved. The discussions will be audio-recorded, and information will be used to develop an interview guide.

Do I have to take part?

No. You decide whether you want to take part. You can stop taking part in the focus group at any time, without reason. Withdrawing from the focus group will not affect your medical care or legal rights. If you withdraw, all personal data will be permanently deleted.

What are the possible risks with taking part?

We do not foresee any disadvantages or risks of taking part in the focus group. However, due to the nature of the symptoms/experiences being discussed the research team appreciate that some questions may be difficult to answer, embarrassing, or cause upset. Participants will be provided support where possible and may skip questions or withdraw their participation. Participants of this focus group will not have to provide personal experiences. Participants may wish to respond to questions that are based only on the design of the interview script. A supportive environment will be maintained, and participants will be able to take as many breaks as needed.

What are the possible benefits of taking part?

We cannot promise the study will help you personally. However, the results should help our understanding of the experience of women with MS and pelvic floor dysfunction. This, in turn, is expected to facilitate the design of a physiotherapeutic programme that targets the direct needs of women with MS. This physiotherapeutic programme will be evaluated during Phase three - a subsequent study, which will be undertaken by the student researcher for completion of their Doctorate award.

What happens after the focus group?

As detailed above, findings from this study will lead to the development of an interview script that will be used in Phase two of this study. After the focus group, there will be a chance for you to register your interest in Phase two. From July 2021, a brief summary of the results from Phase two of the study can be requested from Christine Addington.

What if there is a problem?

If you have any concerns regarding your participation in the study and would like to speak with someone out with the study team, please contact please contact Alexis Henderson (Ethics Research Administrator) on 0141 3313467: alexis.henderson@gcu.ac.uk

Will my taking part in this study be kept confidential?

Yes. Ethical and legal practice will be followed, and all of your information will be handled in confidence. Access to all personal data will be restricted to the research team. Names and email addresses are stored securely and will be destroyed after the study has been completed. The responses that you provide will be treated in confidence. Your rights are protected under the Data Protection Act and any information that might identify you will not be shared outside of the research team. No identifying information will appear in any documents or in the final report. The discussions will be audio-recorded, and the main parts will be transcribed. The audio-recording will be transcribed and safely deleted on the same day. The transcribed discussions will be saved with non-identifiable pseudonyms.

Who has reviewed the study?

The School of Health and Life Sciences departmental committee psychology, social work, and allied health sciences
has granted ethical approval for the study. Consent to take part in the study will be obtained by Christine Addington. You will receive a copy of all signed consent materials.

Further information and contact details

You can get more study information or discuss the project with the student researcher: Christine Addington, caddin200@caledonian.ac.uk

What happens next?

If you decide you are interested in participating in the workshop after reading this information sheet, please contact the student researcher Christine Addington (details above).

Your Rights

Your rights to access, change or move your information are limited, as we need to manage your information in specific ways in order for the research to be reliable and accurate. To safeguard your rights, we will use the minimum personally identifiable information possible.

You can find out more about how we use your information using the following links:
https://www.gcu.ac.uk/dataprotection/
https://www.hra.nhs.uk/information-about-patients/

Alternatively, you can contact the following office:

Data Protection Officer

Department of Governance

Glasgow Caledonian University

Cowcaddens Road

G4 0BA

Tel: 0141 332 8392

dataprotection@gcu.ac.uk

We will be using information from you in order to undertake this focus group and Glasgow Caledonian University will act as the data controller for this study. This data will contain your name, age, contact details and data collected at the focus group. Your personal data will not be shared with any other research bodies.

This means that the Glasgow Caledonian University is responsible for looking after your information and using it properly. Glasgow Caledonian University will keep identifiable information about you for 3 months after the study has finished in a secure location. After this time, all information will be securely destroyed.

Legal basis for processing data

As part of the project we will be recording personal data relating to you. This will be processed in accordance with the General Data Protection Regulation (GDPR); Article 6(1)e. Under GDPR the legal basis for processing your personal data will be the official authority of the university.

Who is organising and funding the study?

This study is being organised by the student researcher and there are no funding bodies associated with this project.

Thank you for taking the time to read this information.

Participant information for interviews

Let's talk pelvic floor... insight into the lived experience of pelvic floor dysfunction in women with multiple sclerosis

Introduction and study purpose

Living with multiple sclerosis (MS) can cause symptoms connected with pelvic floor dysfunction. These symptoms may include urinary or faecal incontinence, issues with bladder emptying, constipation, pelvic organ prolapse, sexual dysfunction and chronic pelvic pain. Physiotherapy has the potential to improve pelvic floor dysfunction. It can improve muscle function, increase confidence and self-esteem, and enhance quality of life. However, for women with MS, current therapy guidelines for pelvic floor dysfunction are inadequate. This study will be one of the first in the UK to ask women with MS about their experiences and needs. The study will consist of three phases:

Phase one: a short online focus group;
Phase two: a private one-to-one interview; and
Phase three: the development of a physiotherapy programme.

You are being invited to take part in **phase two** of the study. Phase two will consist of a short private interview with the student researcher. Based on your preference, this can be either a virtual interview using video call software or telephone. During this interview you have the space to discuss your experiences of pelvic floor dysfunction and its physiotherapeutic management. Your contribution will help us to understand how women experience the impact of pelvic floor dysfunction. The aim is to take the information gained from your interview and develop a MS-centred physiotherapeutic service. This study is being carried out by Christine Addington and is supervised by Professors Doreen McClurg and Suzanne Hagen at Glasgow Caledonian University and Dr Andy Bradshaw at Hull York Medical School.

Before you decide whether to take part, it is important for you to understand what participation in this focus group will involve. Please take time to read the following information carefully and discuss it with others if you wish. Please contact us at the address below if you would like more information.

What will I have to do if I take part?

If you are interested in taking part, you would need access to an electronic device with internet connection that supports video call software or a telephone. You would then be invited to attend a private online video call with the student researcher, Christine Addington. Alternatively, a telephone call can be requested. For this interview we will be using Microsoft Teams a secure video call programme. Details on how to use Microsoft Teams will be provided by email. A test run of the software can be requested to ensure that the connection is ok. The interview will last no longer than 1hr. We will arrange a date and time that is convenient for you. Only you and the student researcher will be present during the interview. Three to five days before your interview you will receive an information pack. This will contain information on pelvic floor dysfunction, current therapies and examples of question that may be asked during your interview. We ask that you have a read through these questions, but it is not necessary to prepare answers. During the interview there will be discussion on your experience of pelvic floor dysfunction, treatment options and challenges. We intend to have only one interview, but it may be necessary to contact you if we need further clarification of responses. The discussions will be audio-recorded and transcribed but will not be identifiable. To capture the experiences of all participants involved in this study, analysis of your response will be done alongside the response of other women with MS.

Do I have to take part?

No. You decide whether you want to take part. You can stop taking part in the interview at any time, without reason. Withdrawing from the interview will not affect your medical care or legal rights. If you withdraw all personal data will be permanently removed.

What are the possible risks with taking part?

We do not foresee any disadvantages or risks of taking part in the interview. But we appreciate that some questions may be difficult to answer, embarrassing, or cause upset. Participants will be provided support where possible and may skip questions or withdraw their participation. We will provide a supportive environment and participants will be able to take as many breaks as needed.

What are the possible benefits of taking part?

We can't promise the study will help you personally. However, the results should help our understanding of pelvic floor dysfunction in women with Multiple Sclerosis. Your input will help us design a therapy service that incorporates the needs of women with MS. This service will be assessed during phase three. Phase three will start late 2021 by the student researcher for completion of their Doctorate award.

What happens after the interview?

As detailed above findings from this study will result in a report that will be used to guide the design of a tailored physiotherapeutic programme. From July 2021, a brief summary of the results from phase two of the study can be requested from Christine Addington.

What if there is a problem?

If you have any concerns regarding your participation in the study and would like to speak with someone out with the study team, please contact please contact Alexis Henderson (ethics research administrator), 0141 3313467: alexis.henderson@gcu.ac.uk

Will my taking part in this study be kept confidential?

Ethical and legal practice will be followed, and all your information will be handled in confidence. Access to all personal data will be restricted to the research team. Names, email addresses and demographic data will be stored securely on an encrypted device. This study will take an audio-recording of your online interview. All recordings will be saved under a pseudonym, there will be no identifiable data attached to the recording. This will be kept for the back-up of data and stored securely on an encrypted laptop. Your responses will be analysed to explore meaningful patterns across the data which will help us to understand the shared or common meanings and/or experiences of the participants. The common themes will be reported in the write up. Confidentiality will not be provided as your answers from the interviews will be written up in the final report; however, anonymity will be guaranteed, and this will occur after the interviews. Meaning that your identity will not be recognisable from your interview data. Your rights are protected under the Data Protection Act and any information that might identify you will not be shared outside of the service evaluation team.

No identifying information will appear in any documents or in the final report.

Who has reviewed the study?

The School of Health and Life Sciences departmental committee psychology, social work, and allied health sciences

has granted ethical approval for the study. Consent to take part in the study will be obtained by Christine Addington. You will receive a copy of all signed consent materials .

Further information and contact details

You can get more study information or discuss the project with the student researcher: Christine Addington, caddin200@caledonian.ac.uk

What happens next?

If you decide you are interested in participating in the workshop, please contact the student researcher Christine Addington (details above).

Your Rights

Your rights to access, change or move your information are limited, as we need to manage your information in specific ways in order for the research to be reliable and accurate. To safeguard your rights, we will use the minimum personally identifiable information possible.

You can find out more about how we use your information using the following links:

https://www.gcu.ac.uk/dataprotection/ https://www.hra.nhs.uk/information-about-patients/

Alternatively, you can contact the following office:

Data Protection Officer

Department of Governance

Glasgow Caledonian University

Cowcaddens Road

G4 0BA

Tel: 0141 332 8392

dataprotection@gcu.ac.uk

We will be using information from you in order to undertake this interview. Glasgow Caledonian University will act as the data controller for this study. This data will contain your

name, age, MS specific data, contact details and data collected at the interview. Your personal data will not be shared with any other third parties.This means that the Glasgow Caledonian University is responsible for looking after your information and using it properly. Glasgow Caledonian University will keep identifiable information about you for 3 months after the study has finished in a secure location. After this time, all information will be securely destroyed.

Legal basis for processing data

As part of the project, we will be recording personal data relating to you. This will be processed in accordance with the General Data Protection Regulation (GDPR); Article 6(1)e. Under GDPR the legal basis for processing your personal data will be the official authority of the university.

Who is organising and funding the study?

This study is being organised by the student researcher and there are no funding bodies associated with this project.

Thank you for taking the time to read this information.
